# Supplementary material for: Faecalibacterium prausnitzii regulates carbohydrate metabolic functions of the gut microbiome in C57BL/6 mice
Source: Gut Microbes. 2025 Jan 22;17(1):2455503. doi: 10.1080/19490976.2025.2455503 (PMC12716062; doi:10.1080/19490976.2025.2455503)
Supplement: Supplemental materials.pdf [file KGMI_A_2455503_SM7911.pdf]

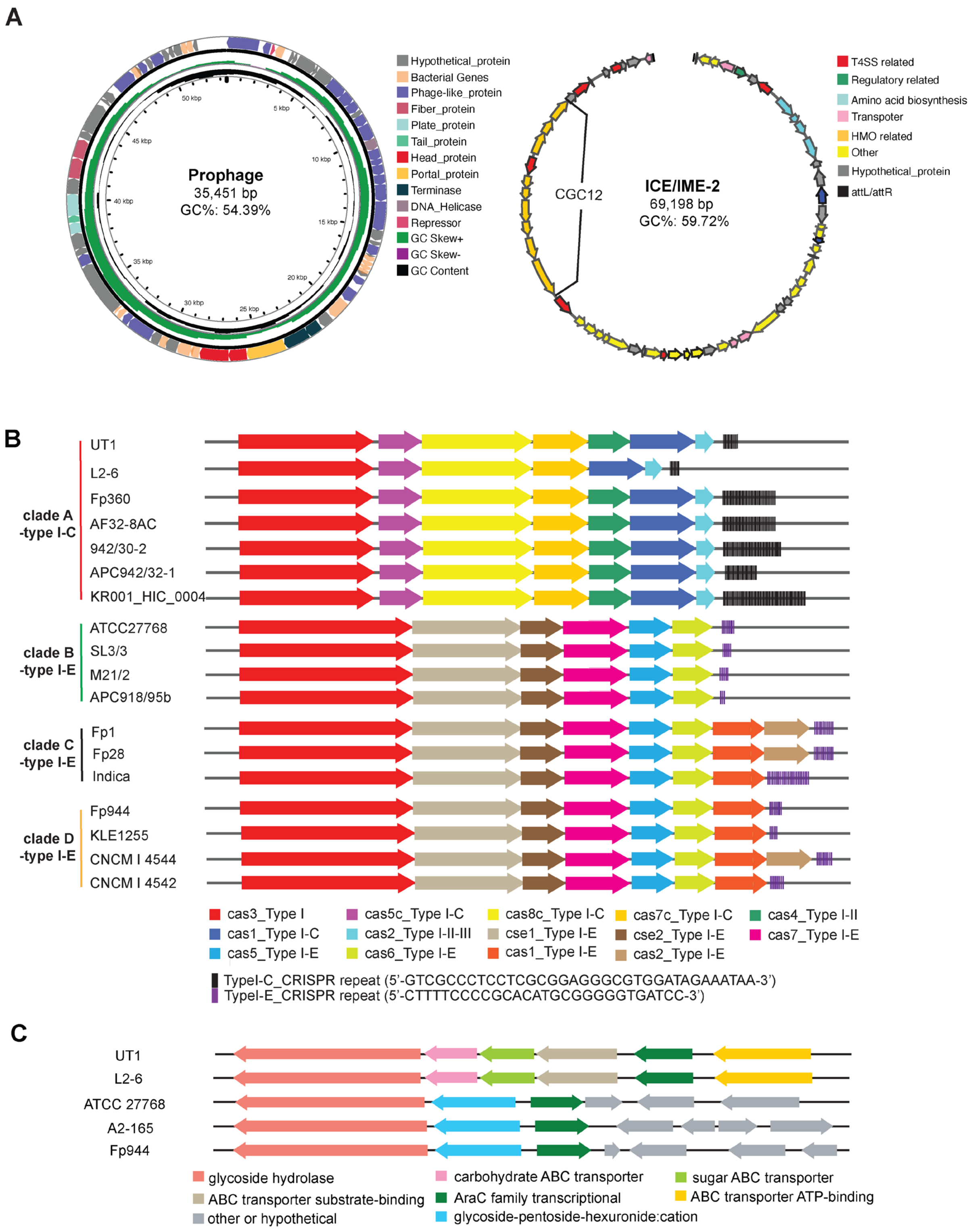

Supplemental Figure 1. Genetic diversity of *F. prausnitzii* strains. (A) Genetic maps of the prophage and ICE/IME-2 in UT1 genome**. (B)** CRISPR-Cas systems predicted in strains of *F.*

prausnitzii clades A, B, C, and D. **(D)** CAZyme gene clusters (CGCs) predicted to utilize galactomannan among strains of different clades.

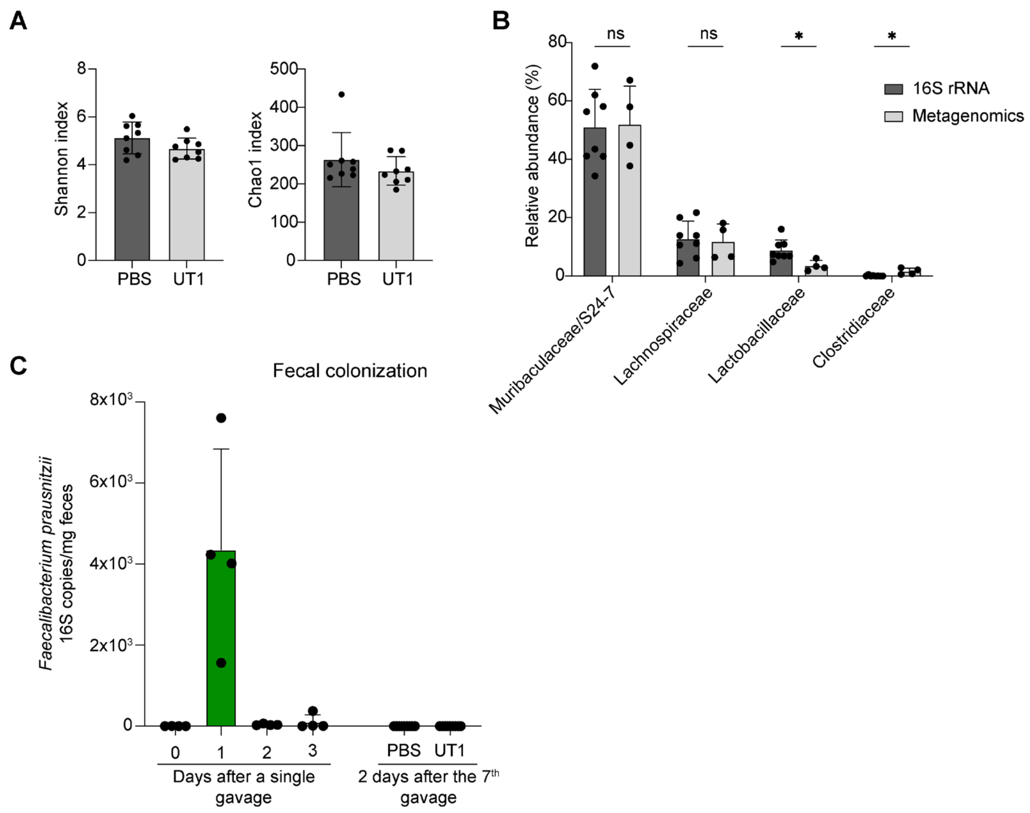

Supplemental Figure 2. Microbiome diversity of UT1-treated mice. (A) Shannon and Chao1 indexes, as measurements of alpha-diversity, obtained from 16S rRNA analysis of UT1- and PBS-gavaged mice. **(B)** Comparison of relative abundances of major bacterial families derived from 16S rRNA and metagenomics analyses. **(C)** C57BL/6 mice (n = 4) were gavaged with *F. prausnitzii* strain UT1 (10^9 CFU/mouse) one time, and fecal samples were collected every day to detect *F. prausnitzii* 16S rRNA copies by qPCR. The 16S copies were also measured in the fecal samples after 7 gavages on day 14 (n=5/group). *$p < 0.05$, unpaired student *t* test.
